# Supplementary material for: Myotoxin-3 from the Pacific Rattlesnake Crotalus oreganus oreganus Venom Is a New Microtubule-Targeting Agent
Source: Molecules. 2022 Nov 25;27(23):8241. doi: 10.3390/molecules27238241 (PMC9739105; doi:10.3390/molecules27238241)
Supplement: Supplementary file 1 [file molecules-27-08241-s001.zip › Legends to Supplementary Files.pdf]

Legends to Supplementary Files

Figure S1: *Bioassay-guided screening of active fractions from* Crotalus oreganus *venom* **A)** HPLC fractions from venom were submitted to tubulin polymerization assay in the presence of DAPI. The increase in fluorescence indicates tubulin polymerization. All the activity was found on fraction B4. **B)** Combined profiles of sub-fractions of B4, loaded separately on analytical HPLC column. **C)** Sub-fractions of B4 were submitted to tubulin polymerization assay in the presence of DAPI. Most of the activity was found in sub-fraction B4-24.

Video S1: *z-stack images from MCF-7 cells transiently transfected with a farnesyl-GFP plasmid to visualize the cell membrane and incubated with Cy3-myotoxin-3*

Video S2: *Dynamic instability of individual microtubules in MCF-7 cells transiently expressing GFP-tagged tubulin (control)*

Video S3: *Dynamic instability of individual microtubules in MCF-7 cells transiently expressing GFP-tagged tubulin (in the presence of 25 µM myotoxin-3)*
