# Supplementary figures and images for: Myotoxin-3 from the Pacific Rattlesnake Crotalus oreganus oreganus Venom Is a New Microtubule-Targeting Agent
Source: Molecules. 2022 Nov 25;27(23):8241. doi: 10.3390/molecules27238241 (PMC9739105; doi:10.3390/molecules27238241)

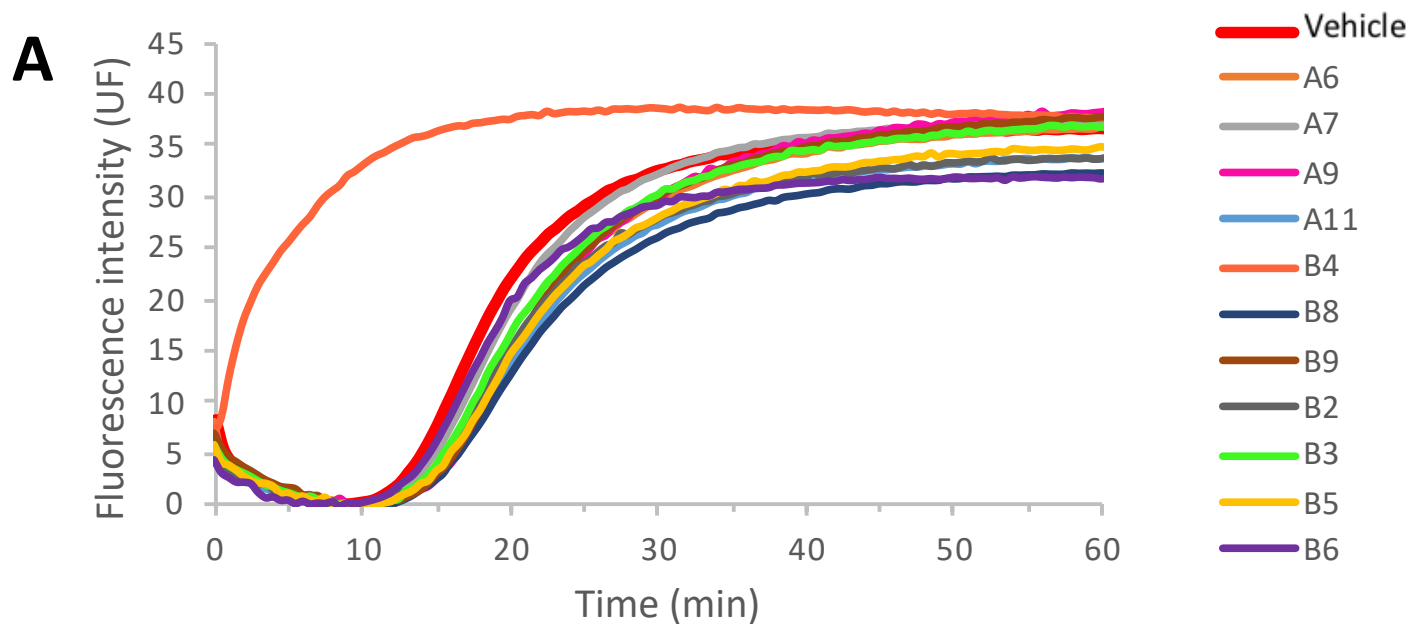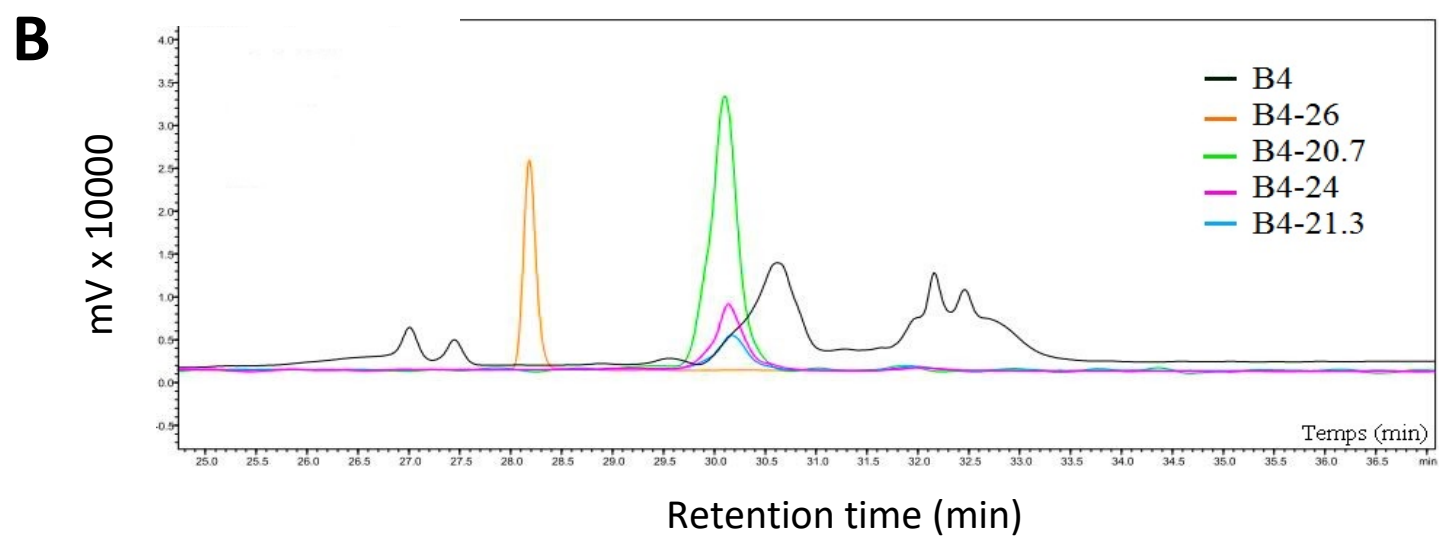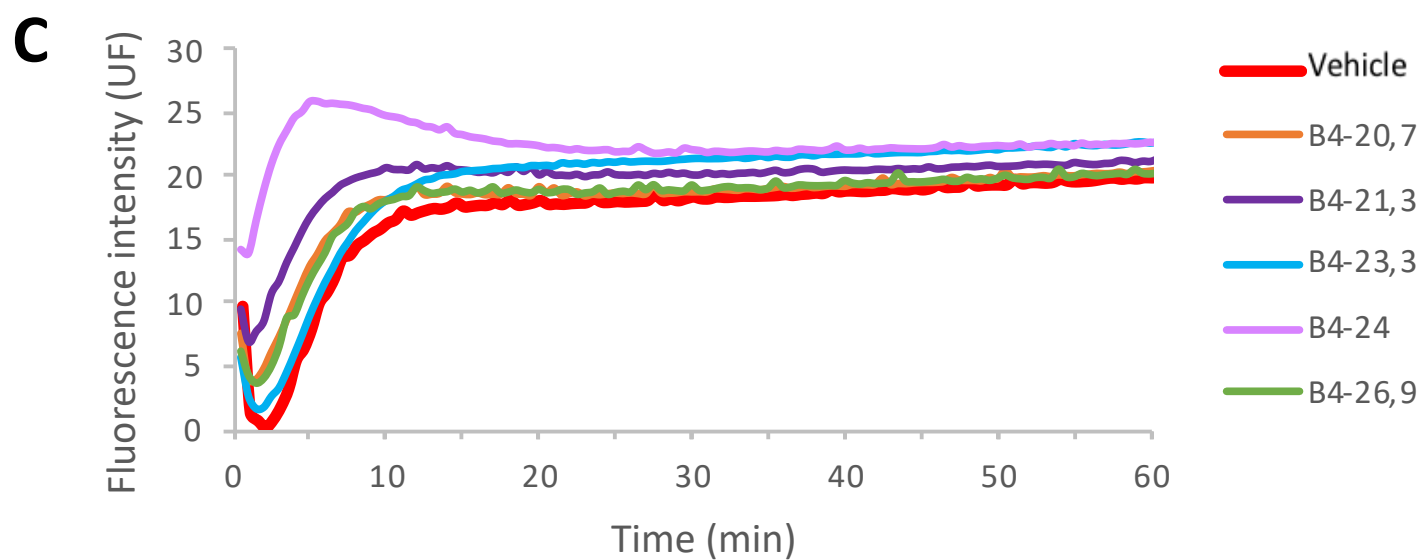

Supplement: Supplementary file 1 [file molecules-27-08241-s001.zip › Figure S1.pdf]
